# Yellow fever re-emergence in Tolima, Colombia 2024–25: an eco-epidemiological study

**DOI:** 10.64898/2026.01.16.26344301

**Published:** 2026-01-19

**Authors:** Seth D. Judson, Nicole Stephan, Sofia Machuca, Andrés F. Henao-Martínez, Gabriel Parra-Henao, Ricardo Vivas, Jose Fair Alarcón-Robayo, Mauricio Javier Vera Soto, Hernán Vargas

**Affiliations:** 1 Division of Infectious Diseases, Department of Medicine, David Geffen School of Medicine at University of California, Los Angeles, USA; 2 Division of Infectious Diseases, Department of Medicine, University of Colorado Anschutz Medical Campus, Aurora, USA; 3 Centro de Investigación en Salud para el Trópico, Universidad Cooperativa de Colombia, Santa Marta, Colombia; 4 Dirección de Salud Pública, Secretaria de Salud del Tolima, Colombia; 5 Subdirección de Enfermedades Transmisibles, Ministerio de Salud y Protección Social, Bogotá, Colombia; 6 Grupo de investigación clínica y medicina traslacional, Hospital Federico Lleras Acosta, Ibagué, Colombia; 7 Grupo de inmunologia molecular, Universidad del Quindío, Armenia, Colombia

## Abstract

**Background:**

Yellow fever virus (YFV) is transmitted by mosquitoes among humans and non-human primates (NHPs) in South America. The 2024–25 yellow fever (YF) outbreak was notable for its spread into new areas, including the department of Tolima in Colombia’s Andean region. We investigated the eco-epidemiology of human and NHP YF cases to understand the patterns and drivers of the Tolima outbreak.

**Methods:**

We collected spatiotemporal, sociodemographic, and mortality data on human YF cases in Tolima, as well as the locations of deceased NHPs with YF. We then conducted exploratory, descriptive, and spatial statistical analyses to identify risk factors and hotspots for YF. We then used inferential spatiotemporal modelling to compare ecological and sociodemographic drivers.

**Findings:**

From September 2024 to October 2025, 116 human and 53 non-human primate YF cases were detected in Tolima. Among the human cases, 77% were male (89/116), and the median age was 47 (interquartile range 34–63). Of these cases, 45 (39%) died, and older age was associated with increased odds of death (adjusted odds ratio, 1.34 per 10 years; 95% confidence interval, 1.10–1.65). There was significant clustering among human and NHP cases. Higher rainfall and poverty/deprivation were associated with increased YF incidence at the neighborhood level, and rainfall during the outbreak was above average due to La Niña.

**Interpretation:**

These findings demonstrate significant mortality from YF, which re-emerged in Tolima after nearly a century. Rural populations with greater ecological risk and poverty/deprivation could benefit from targeted vaccination strategies for YF.

**Funding:**

National Institutes of Health

## Introduction

During 2024–2025, a yellow fever (YF) outbreak in South America expanded into new regions, causing global concern. Yellow fever is a viral hemorrhagic fever caused by a mosquito-borne flavivirus, yellow fever virus (YFV). In South America, the Amazon region is often considered the primary endemic area for YFV circulation. However, in October 2024, YF cases were reported in Tolima, Colombia, a department in the Andean region, which had no known YF cases in the 21st century and low YF vaccination coverage.^1–7^ Concerning features of the 2024–2025 Tolima outbreak included substantial morbidity and mortality, as well as potential peri-urban cases.^4,7^ Understanding the epidemiology and ecology of the YF outbreak in Tolima is essential for coordinating local public health efforts for control and prevention of future outbreaks.

The majority of both historical and recent YF cases in South America have been reported in Brazil, Bolivia, Colombia, Peru, and Ecuador.^8,9^ A significant YF outbreak occurred in Brazil in 2016–2018, notable for the re-emergence of YF in the Atlantic forest region and proximity to major cities. In Colombia, there have been no major YF outbreaks since 2005, aside from sporadic cases.^10^ Thus, the YF outbreak in Tolima is highly concerning and part of a recent trend of YF outbreaks occurring among different ecoregions during the past decade.

Yellow fever virus circulates via two transmission cycles in the Americas—the sylvatic and urban cycles.^8^ The sylvatic cycle involves non-human primate (NHP)-to-mosquito-to-human transmission near forested areas via primarily *Haemagogus* and *Sabethes* mosquitoes. Some new-world NHPs, especially howler monkeys (*Alouatta spp.*), are susceptible to severe disease and mortality from the sylvatic cycle of YFV.^11^ Yellow fever outbreaks in South America have almost exclusively been attributed to the sylvatic cycle since the 1940s, in the context of urban mosquito control and YF vaccination efforts.^7^ Before this there were major outbreaks via the urban cycle, which involves transmission among humans via *Aedes aegypti*. There has been an ongoing resurgence and expansion of *Ae. aegypti* and arboviruses in the Americas, driven by urbanization and environmental change.^12^ The recent YF cases in peri-urban areas of Tolima and São Paulo, Brazil, are concerning for the potential re-establishment of the urban cycle. Thus, understanding the ecological transmission cycle and factors contributing to the YF outbreak in Tolima are important for assessing current and future YF risk.

Given that the severe form of YF has an estimated case fatality ratio (CFR) of 39%^13^, and there are no licensed therapeutics for YF, prevention is key. Understanding the eco-epidemiology of the YF outbreak in Tolima could yield novel insights into risk factors and spatiotemporal patterns of YF, enabling targeted preventive measures such as vaccination and vector control. While multiple hypotheses have been generated surrounding the drivers of the Tolima outbreak^4,5^, spatial statistical and modeling analyses could help understand these factors. Therefore, the goals of our study were to (1) characterize the epidemiology and disease burden of YF in Tolima, (2) analyze spatiotemporal dynamics of YF spread, and (3) identify ecological and sociodemographic risk factors associated with YF incidence.

## Methods

### Setting

The administrative structure of Colombia comprises 32 departments, divided into municipalities, which are further divided into the lowest level, called veredas (neighborhoods). Tolima is a department in Colombia’s central-west region and has 47 municipalities. These municipalities include many rural areas and dispersed settlements, as well as some urban areas such as the capital city, Ibagué.

Tolima has varied topography, including two major branches of the Andes Mountains, the Cordillera Central and Cordillera Oriental, separated by the Magdalena River Valley.^14^ This altitudinal variation contributes to diverse ecosystems in Tolima, including lowland riparian forests, mid-altitude Andean forests, and high-Andean forest and páramos.^14^ Agriculture is the most important economic sector, and Tolima has the third largest rice cultivation area in Colombia.^2^ Agricultural activity in Tolima is associated with deforestation as well as increased seasonal human mobility.^2^ Additionally, there has been an increase in refugees and migration into the region from Venezuela, as well as armed conflict in neighboring departments of Colombia.^2^ A La Niña period began in late 2024 in Colombia, continuing into 2025.

Historical YF outbreaks in Tolima occurred from 1830–1940 (Appendix, Fig. S1).^15,16^ A study of post-mortem human liver samples from 1934–1956 reported YF positive samples from Tolima.^17^ Given the absence of further cases in the region, Tolima was considered a low-risk zone for YF. Consequently, since 2005, routine YF vaccination in Tolima was maintained only for infants at one year of age.

### Human and non-human primate cohort identification

For the 2024–2025 YF outbreak, we conducted a retrospective cohort study of confirmed human YF cases in Tolima, Colombia. We included cases from the earliest confirmed case (based on symptom onset), which occurred September 8^th,^ 2024, to October 31^st,^ 2025. Cases were confirmed at the Laboratorio Nacional de Referencia, del Instituto Nacional de Salud de Colombia (INS) via reverse transcription polymerase chain reaction (RT-PCR). Demographic data, date of symptom onset, mortality status, and location of residence were collected on all cases. Additionally, data were collected by the Corporación Autonoma Regional del Tolima on deceased NHPs in Tolima that were confirmed to have YFV infection post-mortem via RT-PCR and/or histopathology. The locations of these NHPs were collected, including municipalities and veredas, and georeferenced GPS coordinates, when available.

### Statistical Analysis

Descriptive statistics of confirmed human YF cases were calculated in R version 4.5.2. We compared the number of YF cases from Tolima with those reported elsewhere in South America by the Pan American Health Organization (PAHO).^9^ We conducted univariable and multivariable logistic regression to compare the associations between age and sex with mortality status.

To analyze areal-level spatial relationships, we aggregated cases to the municipality and vereda levels. Shapefiles for municipalities and veredas were obtained via Humanitarian Data Exchange under a CC BY-IGO license (https://data.humdata.org/dataset/cod-ab-col). The vereda shapefile was updated in R to address missing areas (Appendix, section 2). Spatial autocorrelation was assessed using global and local versions of Moran’s I, calculated using the spdep package in R. The incidence of YF cases was calculated using WorldPop data aggregated to the municipality and vereda levels.

To analyze point-pattern relationships, the geographic coordinates of residences of confirmed human cases and the locations of infected deceased NHPs were obtained. To identify clustering of cases, the K and L functions were calculated using the spatstat package in R (Appendix, section 4).

### Ecological and Sociodemographic factors

**Figure 1.**
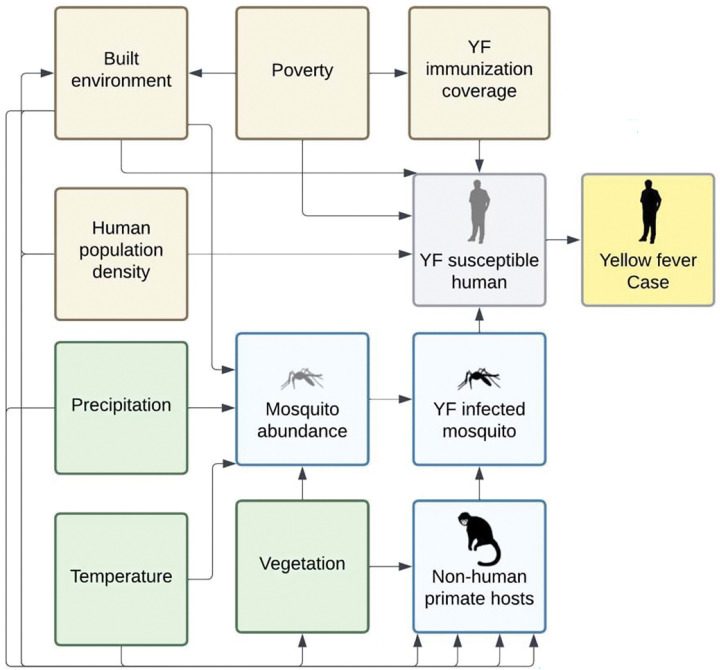
Conceptual framework of potential drivers of yellow fever in Tolima, Colombia The figure depicts key ecological (green) and sociodemographic (brown) variables considered in relation to human yellow fever cases in Tolima. This conceptual framework was used to identify which covariates to include in exploratory spatial analysis and inferential spatiotemporal modelling.

Using a conceptual framework, we identified key covariates potentially associated with YF incidence in Tolima (Figure 1). These covariates were selected based on whether they had previously been associated with YF in other regions, as well as on discussions with YF experts in Tolima.^18–20^ The sources and spatiotemporal resolution for these covariates are shown in Table 1. We included ecological covariates (precipitation, temperature, elevation, vegetation, and land cover) and sociodemographic covariates (population density, poverty/deprivation, and built-up area). We also assessed the species distribution of NHPs in Tolima using the IUCN Redlist.^21^

For our exploratory analysis and modeling, we extracted the mean values for each covariate at the vereda level (Appendix, section 2). We also extracted elevation and land-cover values at specific residence locations. We conducted an initial exploratory statistical analysis by comparing each covariate between veredas that reported YF cases and those that did not (Appendix, section 5).

### Spatiotemporal Modelling Approach

We used a Bayesian inferential modelling approach to identify which covariates were associated with YF incidence in Tolima from 2024 to 2025. First, a correlation matrix was used to exclude covariates that had a correlation coefficient of >|0.7|. There was a strong correlation between temperature and elevation (−0.8) and poverty/deprivation and built-up surface (−0.7), so we elected to keep temperature and poverty/deprivation in the model based on our conceptual framework. We then developed four types of Bayesian Spatiotemporal Hierarchical models using integrated nested Laplace approximation using the R-INLA package. Model I includes fixed effects only. Model II includes fixed effects and spatially structured and unstructured random effects using a Besag-York-Mollié (BYM2) model. Model III adds an unstructured space-time interaction term to capture unexplained temporal variation at the vereda level. Model IV is a full spatiotemporal model that additionally includes a temporally structured random effect, modeled as a first-order random walk. We compared the best fitting of these models to a baseline model with only random effects. Model fit was assessed using the deviance information criterion (DIC) and the Watanabe-Akaike information criterion (WAIC). All covariates were standardized, and we assessed lag values of monthly temperature, precipitation, and NDVI ranging from 1 to 3 months to identify the optimal lag to include in the models. To account for overdispersion, monthly YF cases were modelled as a negative binomial process:

(1) $$Y_{i,t}\sim NegBinom\left(\mu_{i,t}\varphi \right)$$

where φ is the overdispersion parameter and ${Y\;}_{i,t}$ is the observed number of YF cases in vereda i during month t, while $\mu_{i,t}$ is the expected mean number of cases in i at t, modelled in the spatiotemporal model as the log-incidence rate:

(2) $$log\left(\mu_{i,t}\right)=\alpha +log\left(P_{i,t}\right)+X_{i,t}\beta +u_{i}+v_{i}+\omega_{t}+\varepsilon_{i,t}$$

where α is the intercept, $log\left(P_{i,t}\right)$ is an offset to adjust case counts by the logarithm of the population, and X is a matrix of covariates with regression coefficients β. The spatial component follows the Besag-York-Mollié (BYM2) model, where $u_{i}$ is the spatially structured random effect (conditional autoregressive), and $v_{i}$ is the unstructured (i.i.d.) vereda-level random effect. The temporal trend, $\omega_{t}$_,_ is modelled as a first-order random walk, and $\varepsilon_{i,t}$ is an unstructured space-time interaction.

## Results

### Human yellow fever cases

From 2024 to 2025, 116 laboratory-confirmed cases of YF were identified in Tolima (Table 2). Of these cases, 45 (39%) died. These cases represented 85% (116/136) of all YF cases reported in Colombia from January 1^st^, 2024, to October 31^st^, 2025.^9^ Compared with overall YF cases reported in South America during this period, the YF cases in Tolima accounted for 33% (116/356) of all cases, the most of any specific region in South America.^9^

The majority of YF cases were male (77%, 89/116), and the overall median age of cases was 47 [interquartile range (IQR) 34–63]. A higher proportion of deaths occurred among males (83%, 35/42) and those ≥ 60 years old (57%, 24/42). In univariable and multivariable logistic regression analyses, adjusting for sex, age was associated with increased odds of death (adjusted odds ratio, aOR, 1.34 per 10 years; 95% confidence interval, 1.10–1.65) (Appendix, Table S3).

**Figure 2.**
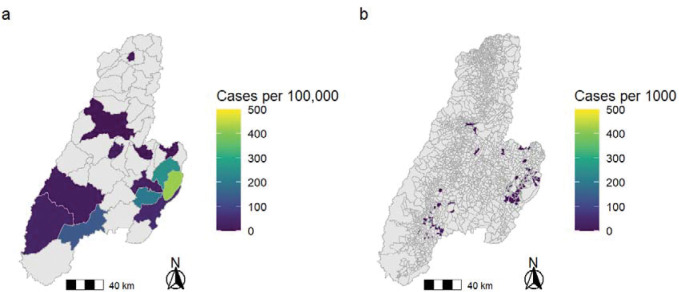
Incidence Rate of Human Yellow Fever Cases in Tolima, Colombia 2024–2025 The incidence rate of human yellow fever cases in Tolima are shown aggregated to the (a) municipality level and (b) vereda level.

The initial YF cases were detected in the southeastern municipalities, with the highest incidence in Cunday, followed by Villarrica, Prado, Purificacion, and Dolores. Subsequently, cases were detected in the central part of the department with single cases in Ibagué, El Espinal, and Valle de San Juan, as well as a single case in the northern municipality of Palocabildo. Multiple YF cases were then detected in the southern municipalities of Ataco, Chaparral, and Rioblanco. There were 74 veredas with YF cases. The incidence of YF cases by municipality and vereda are shown in Figure 2.

### Non-human primate yellow fever cases

Of 53 confirmed NHP cases with locations, 30 individuals had georeferenced coordinates available. The NHP cases occurred in the following municipalities (in order of highest incidence): Chaparral, Ataco, Planadas, Cunday, Purificacion, Villarrica, Rioblanco, San Antonio, and Prado. The genera of NHPs infected included *Alouatta* and *Aotus*.

Based on the IUCN Redlist, NHPs with host ranges in Tolima included: Black-capped Capuchin (*Sapajus apella*), Colombian Red Howler Monkey (*Alouatta seniculus*), Juruá Red Howler Monkey (*Alouatta seniculus subsp. juara*), Colombian Night Monkey (*Aotus lemurinus*), Grey-handed Night Monkey (*Aotus griseimembra*), Varied White-fronted Capuchin (*Cebus versicolor*), and Common Woolly Monkey (*Lagothrix lagothricha*).

### Spatiotemporal analysis

**Figure 3.**
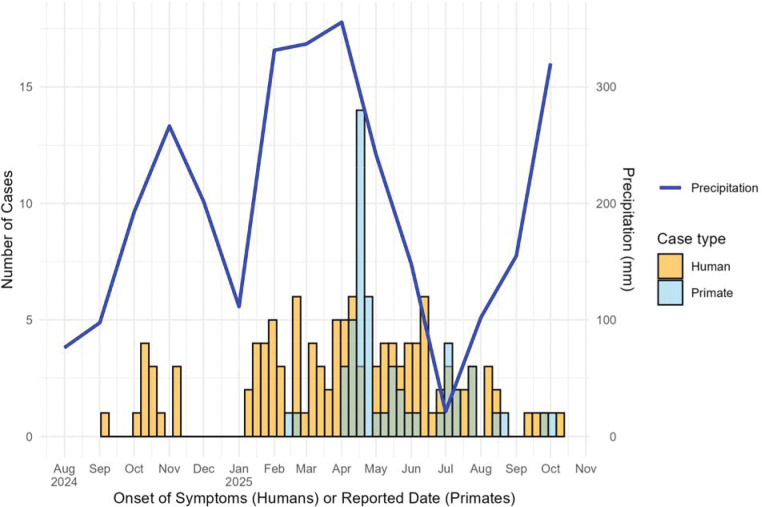
Epidemic Curve of Human and Primate Yellow Fever Cases in Tolima, 2024–2025 The histogram depicts YF cases by week of symptom onset for humans (orange) and reported date of death for NHPs (blue). The line graph shows the overall mean monthly precipitation in Tolima, given the significant association between YF incidence in Tolima and precipitation, as well as the occurrence of La Niña.

Human YF cases occurred between September 8^th^, 2024, and October 8^th^, 2025. The first NHP case was detected on February 10^th^, 2025, and cases continued to be detected until October 1^st^, 2025. The epidemic curve showing the relative timing of human and NHP cases is shown in Figure 3.

Significant spatial autocorrelation among human YF incidence rates was found via Global Moran’s I at the municipality level (*I* = 0.24, p < 0.001), and weaker spatial autocorrelation at the vereda level (*I* = 0.03, p <0.001). Neighboring municipalities and veredas with high YF incidence rates (hotspots) were identified using local Moran’s I (Appendix, section 3). These hotspots occurred in two areas in southern and southeastern Tolima.

**Figure 4.**
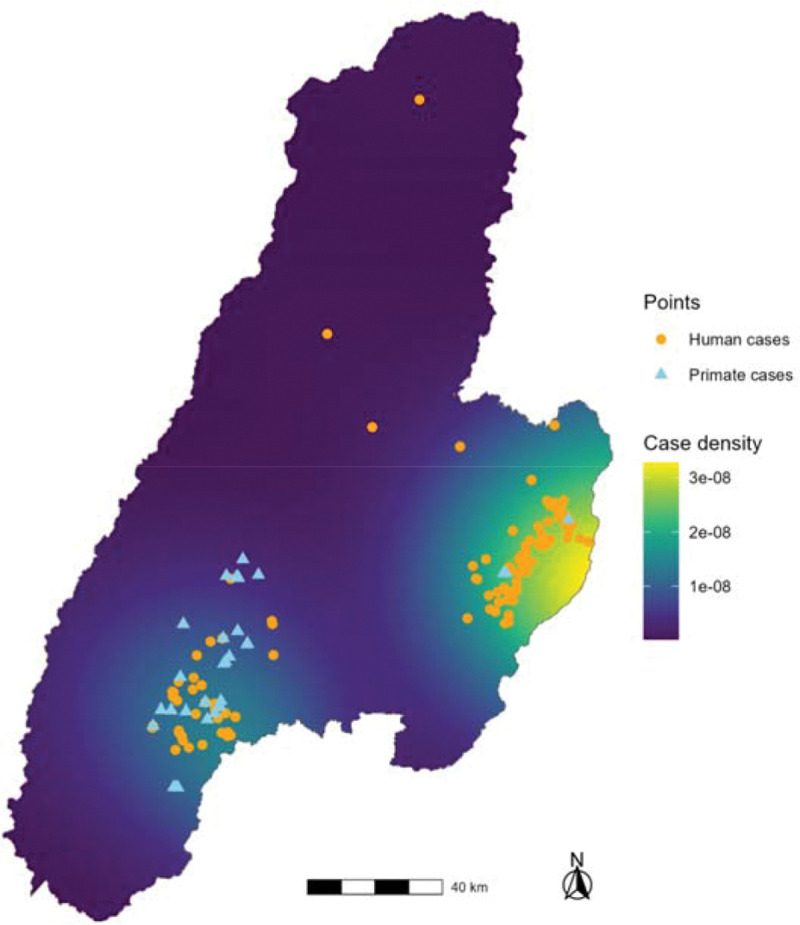
Kernel Density of Human Yellow Fever Cases in Tolima, Colombia- 2024–2025 The georeferenced locations of human and non-human primate yellow fever cases in Tolima are shown. The L function was used to calculate the distance at which maximal human case clustering occurred (23.9 km). Thus, 23 km was selected for the bandwidth of the kernel density estimate to depict the intensity of clustering.

The spatial patterns of human vs NHP YF cases are shown in Figure 4. Human YF cases were found to significantly cluster based on the L function, with clustering strongest at 23.9 km (Appendix, Fig. S4). Non-human primate YF cases were found to form statistically significant clusters with human cases, with clustering strongest at 28 km (Appendix, Fig. S6).

### Exploratory Analysis of Ecological and Sociodemographic Factors

Overall monthly mean precipitation was higher in veredas with YF cases compared to those without cases (p-value <0.001) (Appendix. Fig. S7). The monthly mean precipitation was significantly higher in 8 of 12 months in veredas with YF cases from September 2024–2025 (Appendix, Fig. S8). Precipitation was higher in 9 of 12 months of the study period compared to both the historical 5-year and 30-year averages (Appendix, Fig. S10).

The overall monthly mean temperature was higher in veredas with YF cases compared to those without cases (p-value <0.001) (Appendix, Fig S11). The monthly mean temperature was significantly higher in 12 of 12 months in veredas with YF cases from September 2024 to 2025. Decreased monthly temperature occurred in 12 of 12 months of the study period compared to both historical 5-year and 30-year averages (Appendix, Fig S13).

Yellow fever cases occurred at a mean elevation of 1066 meters (range 318–2190 meters). The mean elevation of veredas with cases was lower than that of those without cases (p = 0.007, Appendix, Fig S15). The highest proportion of YF cases occurred in a mosaic of agriculture and pasture habitat (71%, 82/116), followed by forest (23%, 27/116), infrastructure (3%, 4/116), other non-vegetated area (1%, 1/116), and other non-forest formation (2%, 2/116). Four YF cases occurred in cities/dense towns (including Ibagué) based on urban built-up surface classification. There were no statistically significant differences in overall monthly mean NDVI, population density, poverty/deprivation index, or built-up surface between veredas with YF cases and those without cases (Appendix, Fig. S18, S21, S22).

### Spatiotemporal Modelling Results

Comparing our Bayesian Hierarchical Spatiotemporal modelling results, the spatiotemporal model with standardized covariates and a 3-month lag for precipitation, temperature, and NDVI had optimal performance, as indicated by the lowest WAIC and DIC (Appendix, Table S4). For this model, the incidence rate ratio (IRR) for precipitation was 2.32 (95% credible interval 1.62 – 3.47), temperature 1.25 (95% credible interval 0.79 – 1.98), poverty/deprivation 1.74 (95% credible interval 1.34 – 2.25), and NDVI 1.37 (95% credible interval 0.99 – 1.89). All models identified an increased incidence rate of YF associated with higher mean monthly precipitation and higher mean poverty/deprivation at the vereda level (Appendix, Table S4). The full model had better fit and spatial precision than the baseline model, and structured spatial effects contributed most to the variance (Appendix, Table S5).

## Discussion

The 2024–2025 YF outbreak in Tolima was the largest regional YF outbreak during this period in South America. While YF cases had occurred in Tolima from 1830 to 1940, there were no reported cases in this department for nearly a century. Overall, the epidemiological features of this outbreak are reminiscent of other YF outbreaks in South America, but with key differences. As with other YF outbreaks in South America, the highest proportion of YF cases occurred among middle-aged males, a demographic that includes agricultural workers most likely to be exposed to the sylvatic cycle. However, there was also a high proportion of patients aged 60 years or older with YF (36%), who had a very high CFR (57%), and increased age was found to be significantly associated with mortality. The elderly population was particularly vulnerable during this outbreak because Tolima was considered a low-risk department for YF, and international guidelines advise caution for vaccinating adults aged 60 years or older in low-risk areas due to YF vaccine-associated adverse events.

Additionally, the overall CFR was very high (39%), which is consistent with global estimates for severe YF, indicating that most likely severe YF cases were detected in this cohort, whereas mild or asymptomatic cases were underascertained. This suggests that the actual number of YF cases in Tolima was much higher. The incidence of YF was higher among veredas with greater poverty/deprivation in Tolima, particularly in rural areas. These communities likely have less access to healthcare and lower levels of education. Residents of rural areas are also more likely to be exposed to sylvatic YF vectors due to work activities and proximity to forest borders. Since Tolima was classified as a low-risk department for YF, vaccination remained optional for adults, leading to a significant susceptible population. These findings emphasize the critical importance of immunization for YF (both routine childhood vaccination and mass vaccination campaigns) in areas where YF may circulate, including the Andean region.

We identified clustering of human and NHP cases, particularly around two subsequent foci in the southeastern and southern regions of Tolima. This clustering was strongest between 20 and 30 km for humans and NHPs. *Haemogogus* mosquitoes have a high maximum dispersal distance, estimated at 6 km for *Hg. leucocelaenus* and 11 km for *Hg. janthinomys*.^22^ Additionally, NHPs can act as amplifying hosts, and the interaction between *Haemogogus* mosquitoes and NHPs may may accelerate spread of YFV across ecological corridors.^22^ Therefore, the clusters we detected appear consistent with sequential spread via *Haemogogus* mosquitoes and NHPs in the sylvatic cycle. Additionally, *Haemagogus spp.* are often found on the periphery of forests, and populations increase during higher rainfall. Likewise, higher temperatures shorten the extrinsic incubation period for YFV and could increase vectorial capacity.^23^ We found that precipitation was the strongest environmental driver of YF incidence in the Tolima outbreak. La Niña may have been a key amplifying factor, leading to above-average rainfall during the outbreak. With the predicted increase in intensity and regularity of La Niña, this could increase regional YF risk.^24^

Further considering the ecology of the YF outbreak in Tolima, two primary NHP genera were diagnosed with YF post-mortem (*Alouatta* and *Aotus*). The majority of YF cases occurred among *Alouatta seniculus*, which are highly susceptible to YF and exhibit high mortality. If *Alouatta spp.* are infected, they either die or develop immunity, thus acting as sentinels for YF outbreaks.^11^ *Alouatta spp.* are widespread and are able to survive in fragmented landscapes, including Andean forests that have undergone deforestation.^25^ *Aotus spp.* also often develop fatal infection and high viremia with YFV and can live in peri-urban forest fragments.^11,26,27^ Therefore both *Alouatta* and *Aotus* may have been involved in amplifying the Tolima outbreak. Other species present in Tolima include *Sapajus apella*, *Cebus versicolor*, and *Lagothrix lagothricha,* which are thought to develop less severe infection from YFV. However, both *Cebus* and *Lagothrix* were recently found to develop fatal YFV infection under captive conditions in Colombia.^28^ Multiple epizootic YF outbreaks among NHPs were reported during 2024–2025, including the department of Huila which borders Tolima.^29^ Given that there was not a national surveillance system for YF in NHPs in Colombia, deceased NHPs were tested in response to the YF outbreak in humans, making it difficult to assess the temporality between human and NHP cases.

The majority of YF cases occurred in rural areas near forested habitats. Initial cases occurred near Bosque de Galilea Natural Park among individuals involved in agricultural and logging activities in the forest. Deforestation could increase habitat fragmentation for NHPs and bring humans into closer contact with YFV-infected NHPs and vectors. Such forest fragmentation has been considered to be a driver of YF spread in other regions.^30^

Overall, the epidemiology and ecology of this outbreak strongly support the sylvatic transmission cycle. However, there were four individual cases near urban areas, including one in the capital city of Ibagué. To fully assess the risk of urban transmission, additional epidemiological and entomological analyses are needed to determine where these peri-urban cases most likely acquired infection.

There are multiple limitations to our analysis. We used the locations of residence of human YF cases as a proxy for where infection occurred. To compare covariates with YF incidence and protect privacy, we aggregated cases to the areal level. This can lead to the Modifiable Areal Unit Problem (MAUP), a bias introduced by aggregating continuous geographic variables into discrete areas. However, we minimized this by aggregating to the vereda level, the smallest administrative unit with a mean area of 12 km^2^ per vereda (Appendix). While all covariates were aggregated to the vereda level for our analysis, monthly precipitation and temperature were at a coarser spatial resolution than the other covariates. Local downscaling techniques that account for cloud cover would be needed to obtain these covariates at higher resolution. While we were able to compare multiple factors from our conceptual framework, we were unable to directly assess the role of YF vaccination coverage due to a lack of granular data. In the future, including vaccination coverage, as well as data on human mobility (transhumance) and entomologic surveillance could help improve our understanding of transmission dynamics.

In summary, the re-emergence of YF in Tolima revealed critical lessons regarding YF eco-epidemiology and prevention. While this region had not experienced YF for nearly a century, it had the appropriate ecological conditions for the spread of sylvatic YF. These conditions, likely amplified by La Niña, and a rural, unvaccinated population with high levels of poverty and deprivation, fueled a substantial YF outbreak. Identifying other regions with similar ecological and sociodemographic risk factors is essential for targeted YF vaccination campaigns to prevent future outbreaks.

## Supplementary Material

Supplement 1

## Figures and Tables

**Table 1.** Ecological and Sociodemographic Covariates

Covariate	Details	Resolution	Source
Monthly mean precipitation 2024–2025	Satellite-based thermal infrared rainfall estimates with in-situ station observations	0.05°	CHIRPS v3
Monthly mean temperature 2024–2025	Satellite-based land surface temperature	0.05°	MODIS/061/MOD21C3
Elevation	Derived from the Shuttle Radar Topography (SRTM) elevation data	30 arc seconds/1 km^2^	Worldclim 2.1
Monthly Vegetation (NDVI) 2024–2025	Normalized Difference Vegetation Index	1 km^2^	MODIS/061/MOD13A3
Annual Landcover 2024	Satellite-based with machine learning landcover classification_	30 m^2^	MapBiomas
Population density 2025	Gridded population estimates	100 m^2^	Worldpop
Deprivation and Poverty 2020	Weight indices of sociodemographic and satellite data	1 km^2^	Global Gridded Relative Deprivation Index, Version 1
Built-up surface area 2025	Built-up surface based on Sentinel Earth Observation data	100 m^2^	Copernicus Global Human Settlement Layer

**Table 2.** Yellow Fever Cases in Tolima, Colombia, September 2024 – October 2025

	Confirmed Death (N=42)	Confirmed Alive (N=74)	Overall (N=116)

**Age**			
Median [IQR]	62 [39, 70]	44 [28, 59]	47 [34, 63]
**Age Group**			
Age 0 –18	0 (0%)	10 (13.5%)	10 (8.6%)
Age 19–39	11 (26.2%)	21 (28.4%)	32 (27.6%)
Age 40–59	7 (16.7%)	25 (33.8%)	32 (27.6%)
Age ≥ 60	24 (57.1%)	18 (24.3%)	42 (36.2%)
**Sex**			
F	7 (16.7%)	20 (27.0%)	27 (23.3%)
M	35 (83.3%)	54 (73.0%)	89 (76.7%)

## Data Availability

The individual line-level human data from this study contain protected health information and are restricted from public sharing. These data may be made available upon request, subject to approval from the Secretaría de Salud del Tolima, Colombia, and from institutional review boards. Municipality and vereda-level aggregate human data and NHP data from this study and R scripts are available at: https://doi.org/10.6084/m9.figshare.31061575 and https://github.com/Judson-Lab/YF_Tolima
